# Supplementary figures and images for: Functional Blockage of S100A8/A9 Ameliorates Ischemia–Reperfusion Injury in the Lung
Source: Bioengineering (Basel). 2022 Nov 10;9(11):673. doi: 10.3390/bioengineering9110673 (PMC9687586; doi:10.3390/bioengineering9110673)

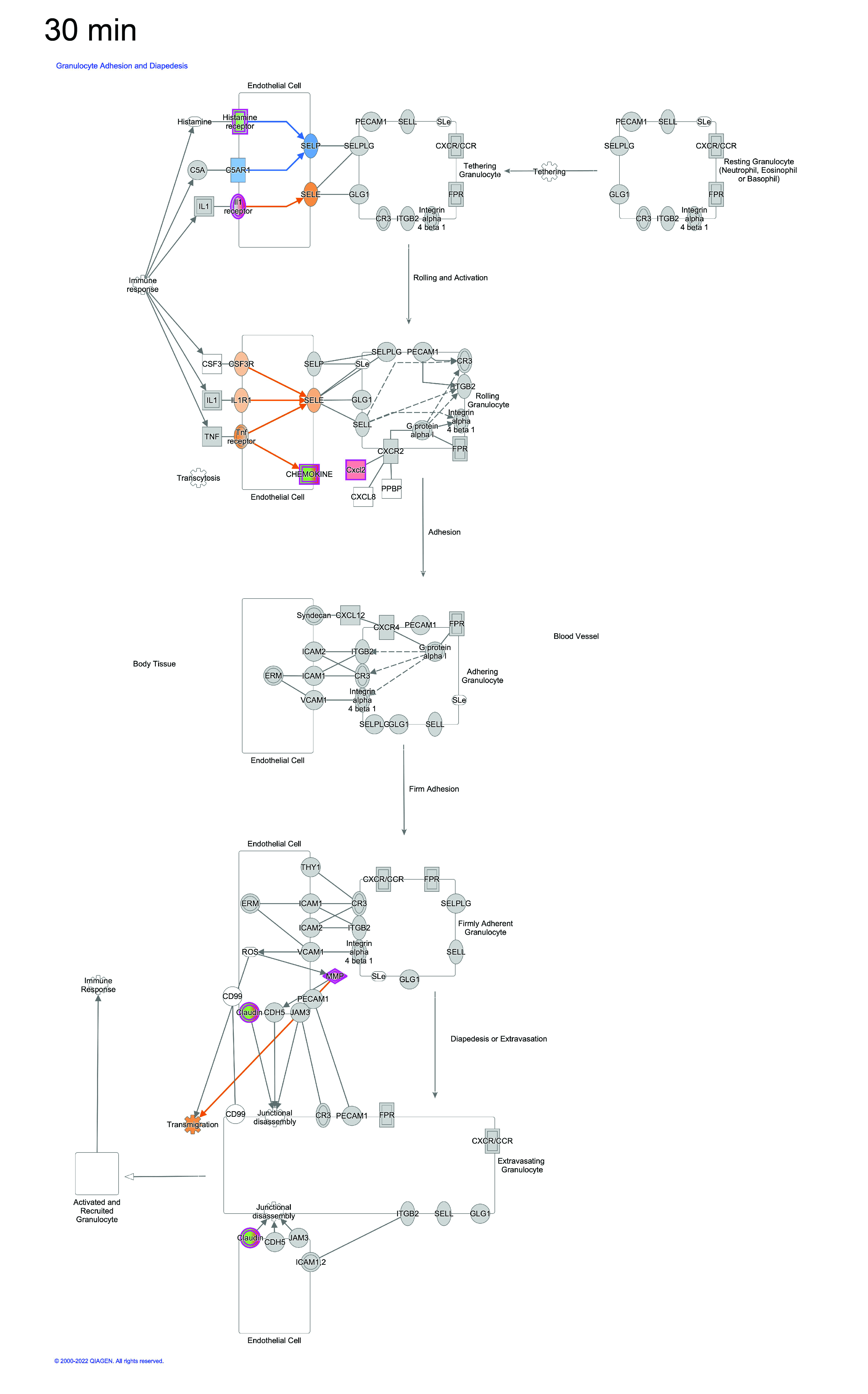

Supplement: Supplementary file 1 [file bioengineering-09-00673-s001.zip › Figure S1.jpg]

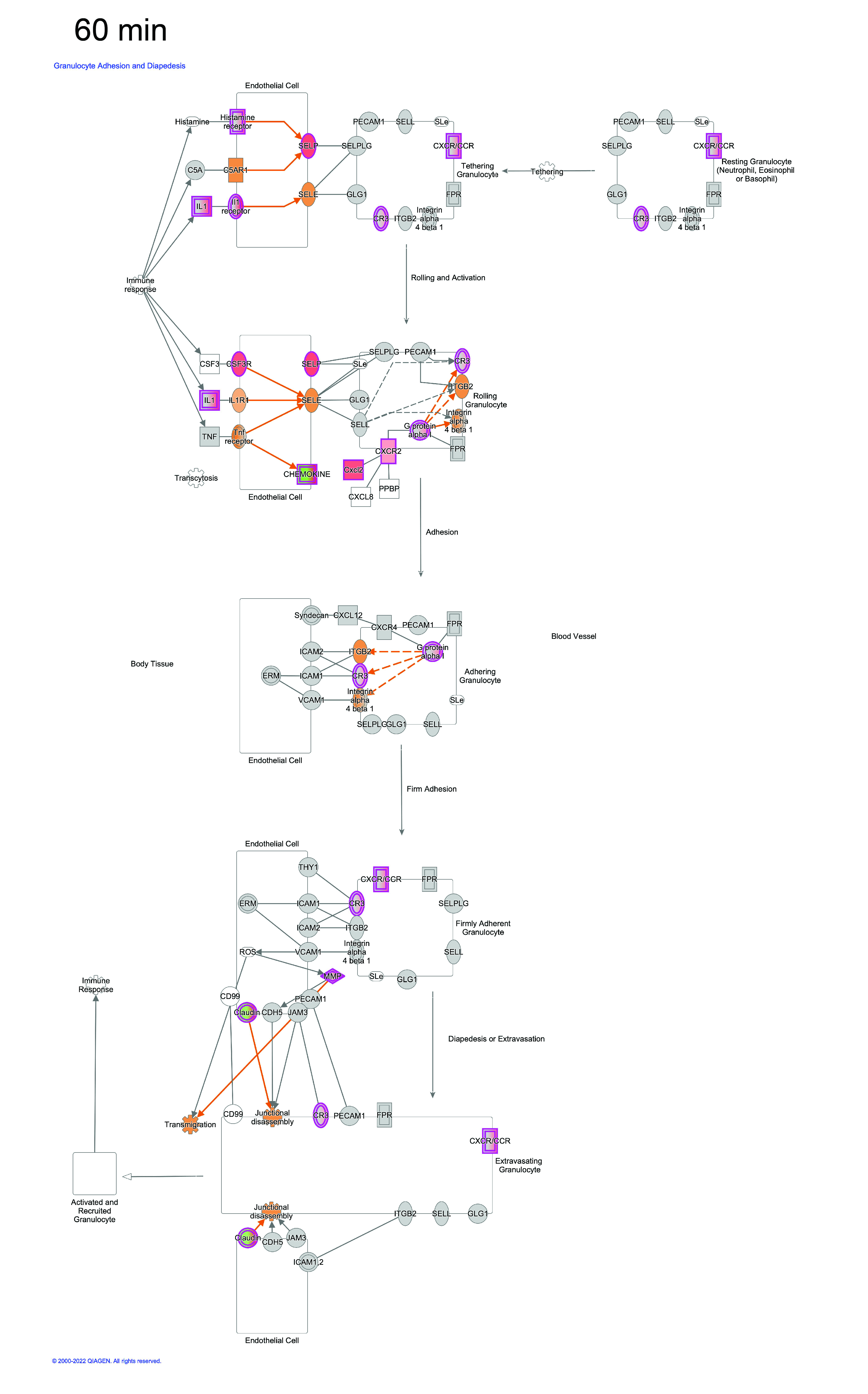

Supplement: Supplementary file 1 [file bioengineering-09-00673-s001.zip › Figure S2.jpg]

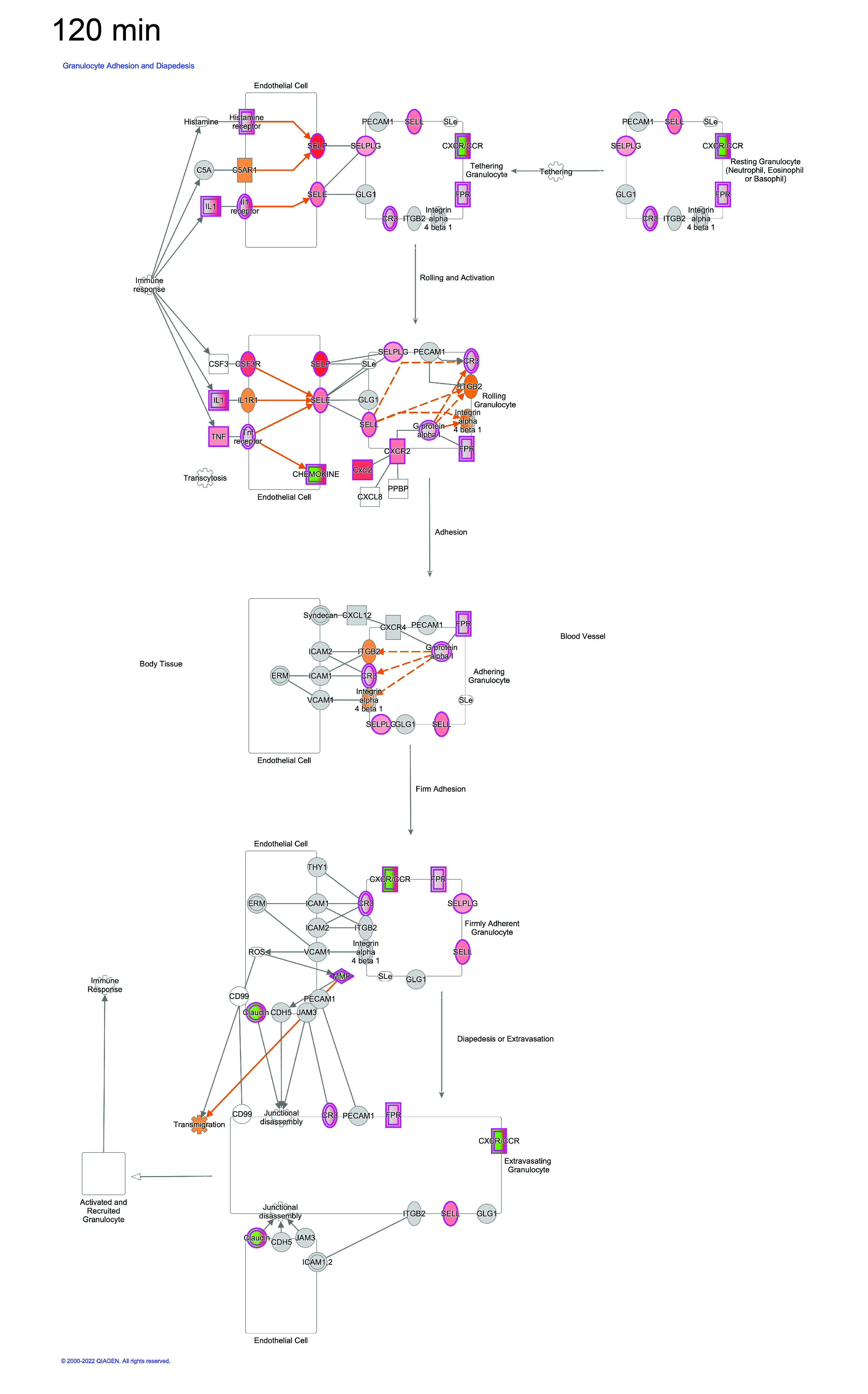

Supplement: Supplementary file 1 [file bioengineering-09-00673-s001.zip › Figure S3.jpg]
